# Supplementary material for: Uncovering the Biotechnological Importance of Geotrichum candidum
Source: Foods. 2023 Mar 7;12(6):1124. doi: 10.3390/foods12061124 (PMC10048088; doi:10.3390/foods12061124)
Supplement: Supplementary file 1 [file foods-12-01124-s001.zip › foods-2196504-supplementary.pdf]

Supplementary Table S1. Identified enzymes in *G. candidum* genome, their EC number, sequence length and preliminary catalytic activities

Name of enzyme / EC number	Biological process	UniProtKB entry	Sequence length (amino acids)	Preliminary catalytic activities
Ubiquinone biosynthesis monooxygenase COQ6, mitochondrial / EC:1.14.13	Lytic polysaccharide monooxygenases	A0A0J9X5N2_GEOCN	505	4-hydroxy-3-all-trans-hexaprenylbenzoate + 2 H+ + O2 + 2 reduced [2Fe-2S]-[ferredoxin] = 3,4-dihydroxy-5-all-trans-hexaprenylbenzoate + H2O + 2 oxidized [2Fe-2S]-[ferredoxin]
Deoxyhypusine hydroxylase EC:1.14.99.29		A0A0J9XE94_GEOCN	313	[eIF5A protein]-deoxyhypusine + AH2 + O2 = [eIF5A protein]-hypusine + A + H2O
5-demethoxyubiquinone hydroxylase, mitochondrial / EC:1.14.99.60		A0A0J9XB16_GEOCN	201	a 6-methoxy-3-methyl-2-all-trans-polyprenyl-1,4-benzoquinol + AH2 + O2 = A + a 3-demethylubiquinol + H2O
Kynurenine 3-monooxygenase / EC:1.14.13.9		A0A0J9XJC8_GEOCN	523	H+ + L-kynurenine + NADPH + O2 = 3-hydroxy-L-kynurenine + H2O + NADP+
Squalene monooxygenase / EC:1.14.14.17		A0A0J9XB44_GEOCN	467	O2 + reduced [NADPH--hemoprotein reductase] + squalene = (S)-2,3-epoxysqualene + H+ + H2O + oxidized [NADPH--hemoprotein reductase]
Choline monooxygenase, chloroplastic / EC:1.14.15.7		A0A0J9XB73_GEOCN	446	choline + 2 H+ + O2 + 2 reduced [2Fe-2S]-[ferredoxin] = betaine aldehyde hydrate + H2O + 2 oxidized [2Fe-2S]-[ferredoxin]
Flavin-containing monooxygenase / UniProtKB unreviewed (TrEMBL)		A0A0J9XBF3_GEOCN / A0A0J9XHW9_GEOCN / A0A0J9X4X4_GEOCN	515 / 518 / 453	Bifunctional peptidase and (3S)-lysyl hydroxylase
Lanosterol 14-alpha-demethylase / UniProtKB unreviewed (TrEMBL)		A0A0J9X4F9_GEOCN / A0A0J9XJ09_GEOCN	539 / 538	steroid metabolic process
Sphingolipid delta(4)-desaturase / 1.14.19.17		A0A0J9XIK7_GEOCN	357	Delta4-fatty-acid desaturase which introduces a double bond at the 4-position in the long-chain base (LCB) of ceramides
Delta 8-(E)-sphingolipid desaturase / EC:1.14.19.18		A0A0J9XF15_GEOCN	579	Delta8-fatty-acid desaturase which introduces a double bond at the 8-position in the long-chain base (LCB) of ceramides. Required for the formation of the di-unsaturated sphingoid base (E,E)-sphinga-4,8-dienine during glucosylceramide (GluCer) biosynthesis.
Delta 12 fatty acid desaturase / UniProtKB unreviewed (TrEMBL)		A0A2S0VYJ3_GEOCN	412	lipid metabolic process
fatty acid desaturase / UniProtKB unreviewed (TrEMBL)		A0A0J9X484_GEOCN	529	Desaturation of oleic acid and linoleic acid (LA) to produce LA and α-linolenic acid
sterol desaturase / UniProtKB unreviewed (TrEMBL)		A0A0J9XE52_GEOCN	375	introduction of a C-5(6) double bond into episterol, a in ergosterol biosynthesis

Endo-polygalacturonase S31PG1 / UniProtKB unreviewed (TrEMBL)	Q96WQ0_GEOCN / Q96WQ1_GEOCN	369 / 368	pectin catabolic process
Polygalacturonase / UniProtKB unreviewed (TrEMBL)	Q874F0_GEOCN	368	pectin catabolic process
Endo-polygalacturonase Ap2PG1 / UniProtKB unreviewed (TrEMBL)	Q8NK97_GEOCN	366	pectin catabolic process
Endo-beta-1,4-glucanase D / EC:3.2.1.4	A0A0J9XK58_GEOCN / A0A0J9XL55_GEOCN	530 / 359	Endohydrolysis of (1->4)-beta-D-glucosidic linkages in cellulose, lichenin and cereal beta-D-glucans
endo-1,3(4)-beta-glucanase / EC:3.2.1.6	A0A0J9X5E1_GEOCN / A0A0J9XD54_GEOCN	974 / 718	Endohydrolysis of (1->3)- or (1->4)-linkages in beta-D-glucans when the glucose residue whose reducing group is involved in the linkage to be hydrolyzed is itself substituted at C-3
Endo-beta-1,3-glucanase / UniProtKB unreviewed (TrEMBL)	A0A0J9X4U5_GEOCN	308	carbohydrate metabolic process, cell wall maintenance
Cellulase / EC:3.2.1.4	A0A0J9X4K1_GEOCN / A0A0J9XBN8_GEOCN /A0A0J9XD87_GEOCN / A0A0J9X5P0_GEOCN	337 / 506 / 407 / 516	Endohydrolysis of (1->4)-beta-D-glucosidic linkages in cellulose, lichenin and cereal beta-D-glucans
Glucanase / UniProtKB unreviewed (TrEMBL)	A0A0J9XBN3_GEOCN / A0A0J9X4S0_GEOCN / A0A0J9X4Q1_GEOCN / A0A0J9XHP1_GEOCN	346 / 376 / 428 / 572	Cellulose degradation
Major exo-1,3-beta-glucanase / UniProtKB unreviewed (TrEMBL)	A0A0J9X636_GEOCN / A0A0J9XAN8_GEOCN	420 / 969	hydrolyze O-glycosyl compounds
Putative glycosidase / UniProtKB unreviewed (TrEMBL)	A0A0J9XJI1_GEOCN / A0A0J9X441_GEOCN	728 / 778	lipid catabolic process, hydrolyze O-glycosyl compounds
glycoside hydrolase / UniProtKB unreviewed (TrEMBL)	A0A0J9XIM3_GEOCN / A0A0J9XEP7_GEOCN	528 / 515	Cellulose degradation
Glycogen debranching enzyme / EC:3.2.1.33	A0A0J9XHF4_GEOCN	1585	Hydrolysis of (1->6)-alpha-D-glucosidic branch linkages in glycogen phosphorylase limit dextrin.
alpha-mannosidase / EC:3.2.1.24	A0A0J9XFS7_GEOCN	1111	Hydrolysis of terminal, non-reducing alpha-D-mannose residues in alpha-D-mannosides
chitinase / EC:3.2.1.14	A0A0J9XGX6_GEOCN / A0A0J9X4I7_GEOCN / A0A0J9XF26_GEOCN / A0A0J9XJG2_GEOCN / A0A0J9X9V6_GEOCN / A0A0J9XBA8_GEOCN	598 / 848 / 673 / 852 / 301 / 428	Random endo-hydrolysis of N-acetyl-beta-D-glucosaminide (1->4)-beta-linkages in chitin and chitodextrins
beta-N-acetylhexosaminidase / EC:3.2.1.52	A0A0J9XF75_GEOCN	719	Hydrolysis of terminal non-reducing N-acetyl-D-hexosamine residues in N-acetyl-beta-D-hexosaminides
1,4-alpha-glucan-branching enzyme / EC:2.4.1.18	A0A0J9XAK6_GEOCN	683	Transfers a segment of a (1->4)-alpha-D-glucan chain to a primary hydroxy group in a similar glucan chain
1,3-beta-glucanosyltransferase / EC:2.4.1	A0A0J9X2R6_GEOCN / A0A0J9X6X0_GEOCN / A0A0J9XH30_GEOCN /	466 / 464 / 496 /	Splits internally a 1,3-beta-glucan molecule and transfers the newly generated reducing end (the donor) to

		A0A0J9X4I4_GEOCN / A0A0J9XEC8_GEOCN / A0A0J9X4U7_GEOCN	514 /557 / 553	the non-reducing end of another 1,3-beta-glucan molecule (the acceptor) forming a 1,3-beta linkage, resulting in the elongation of 1,3-beta-glucan chains in the cell wall
Glycogen debranching enzyme / EC:3.2.1.33, EC:2.4.1.25		A0A0J9X3H4_GEOCN	1566	Multifunctional enzyme acting as 1,4-alpha-D-glucan:1,4-alpha-D-glucan 4-alpha-D-glycosyltransferase and amylo-1,6-glucosidase in glycogen degradation
Mannosyl-oligosaccharide glucosidase / EC:3.2.1.106		A0A0J9X694_GEOCN	827	Cleaves the distal alpha 1,2-linked glucose residue from the Glc3Man9GlcNAc2 oligosaccharide precursor
Glyco_hydro_3 domain-containing protein / UniProtKB unreviewed (TrEMBL)		A0A0J9X3Q8_GEOCN / A0A0J9XFN5_GEOCN	1085 /1125	carbohydrate metabolic process
Glucosidase II catalytic subunit / UniProtKB unreviewed (TrEMBL)		A0A0J9X846_GEOCN	945	carbohydrate metabolic process
Chitin deacetylase / UniProtKB unreviewed (TrEMBL)		A0A0J9XCB5_GEOCN / A0A0J9X6M1_GEOCN	503 / 366	carbohydrate metabolic process
lipase 1 / EC:3.1.1.3	Lipases	P17573 · LIP1_GEOCN	563	a triacylglycerol + H2O = a diacylglycerol + a fatty acid + H+
Lipase 2 / EC:3.1.1.3		P22394 · LIP2_GEOCN	563	a triacylglycerol + H2O = a diacylglycerol + a fatty acid + H+
Bifunctional enzyme with triacylglycerol lipase and lysophosphatidylethanolamine acyltransferase activity / / UniProtKB unreviewed (TrEMBL)		A0A0J9X5Q2_GEOCN	601	triglyceride metabolic process
triacylglycerol lipase / EC:3.1.1.3		A0A0J9X3H5_GEOCN / A0A0J9X719_GEOCN / A0A0J9YHF8_GEOCN	359 / 949 / 521	lipid catabolic process
Hormone-sensitive lipase / UniProtKB unreviewed (TrEMBL)		A0A0J9X8B2_GEOCN	573	lipid catabolic process
Lipase_3 domain-containing protein / UniProtKB unreviewed (TrEMBL)		A0A0J9XB09_GEOCN	550	lipid catabolic process
Carboxylic ester hydrolase / UniProtKB unreviewed (TrEMBL)		Q12616_GEOCN / Q2PPA8_GEOCN / Q1ZZV0_GEOCN / A0A0D4D445_GEOCN / A0A0J9X5E4_GEOCN / A0A0J9X5C5_GEOCN / A0A0P0CZC3_GEOCN / Q0MVP3_GEOCN / D0F1S1_GEOCN	563 / 563 / 544 / 563 / 563 / 530 / 563 / 563 / 563	lipid catabolic process
Oleic acid-inducible, peroxisomal matrix localized lipase / UniProtKB unreviewed (TrEMBL)		A0A0J9X3T0_GEOCN / A0A0J9X7S6_GEOCN	392 / 376	lipid catabolic process
Patatin-like phospholipase		A0A0J9XIA0_GEOCN	691	triglyceride metabolic process

domain-containing protein / EC:3.1.1				
Carboxylic ester hydrolase / EC:3.1.1.3		A0A076U4Z7_GEOCN / Q12614_GEOCN	517 / 544	a triacylglycerol + H2O = a diacylglycerol + a fatty acid + H+
DUF676 domain-containing protein / UniProtKB unreviewed (TrEMBL)		A0A0J9X669_GEOCN / A0A0J9XH06_GEOCN	460 / 479	lipid catabolic process
COesterase domain-containing protein / UniProtKB unreviewed (TrEMBL)		A0A0J9X675_GEOCN	569	carboxylesterase_lipase
Carboxylic ester hydrolase / UniProtKB unreviewed (TrEMBL)		A0A0D3QFR9_GEOCN	544	lipid catabolic process
Steryl ester hydrolase / UniProtKB unreviewed (TrEMBL)		A0A0J9X6L7_GEOCN	540	lipid metabolic process
ATP-dependent serine protease / EC:3.4.21.53	Proteases	A0A0J9X4P2_GEOCN / A0A0J9XHM4_GEOCN / A0A0J9XEH2_GEOCN	1026 / 928 / 1082	Hydrolysis of proteins in presence of ATP.
Cysteine protease / EC:3.4.22		A0A0J9X440_GEOCN	609	[protein]-C-terminal L-amino acid-glycyl-phosphatidylethanolamide + H2O = [protein]-C-terminal L-amino acid-glycine + a 1,2-diacyl-sn-glycero-3-phosphoethanolamine
Aspartic protease / UniProtKB unreviewed (TrEMBL)		M1XFD0_GEOCN	520	aspartic-type endopeptidase activity
CAAX prenyl protease / EC:3.4.24.84		A0A0J9X2L8_GEOCN	448	Proteolytically removes the C-terminal three residues of farnesylated proteins
ULP_PROTEASE domain-containing protein / UniProtKB unreviewed (TrEMBL)		A0A0J9X4C9_GEOCN	384	deNEDDylase activity
Vacuolar aspartyl protease (Proteinase A) / UniProtKB unreviewed (TrEMBL)		A0A0J9XEH7_GEOCN / A0A0J9X3D7_GEOCN	569 / 397	Aspartyl protease
Subtilisin-like protease / UniProtKB unreviewed (TrEMBL)		A0A0J9X5E7_GEOCN / A0A0J9X3G9_GEOCN / A0A0J9X633_GEOCN / A0A0J9XFX0_GEOCN / A0A0J9XHJ8_GEOCN / A0A0J9YHD6_GEOCN	911 / 401 / 369 / 525 / 473 / 475	serine-type endopeptidase activity
Vacuolar aspartyl protease (Proteinase A) / UniProtKB unreviewed (TrEMBL)		A0A0J9XES0_GEOCN	517	aspartic-type endopeptidase activity
Putative GPI-anchored aspartic protease / UniProtKB unreviewed (TrEMBL)		A0A0J9X7Z5_GEOCN	647	aspartic-type endopeptidase activity
Ulp1 protease family protein / UniProtKB unreviewed (TrEMBL)		A0A0J9XBS1_GEOCN	1075	protease

Leukotriene A(4) hydrolase / EC:3.3.2.10	A0A0J9XKH8_GEOCN	664	Aminopeptidase that preferentially cleaves di- and tripeptides. Also has low epoxide hydrolase activity (in vitro). Can hydrolyze the epoxide leukotriene LTA4 but it forms preferentially 5,6-dihydroxy-7,9,11,14-eicosatetraenoic acid rather than the cytokine leukotriene B4 as the product compared to the homologous mammalian enzyme (in vitro)
Peroxisomal leader peptide-processing protease / UniProtKB unreviewed (TrEMBL)	A0A0J9XAX9_GEOCN	544	serine-type endopeptidase activity
rhomboid protease/ UniProtKB unreviewed (TrEMBL)	A0A0J9XEI2_GEOCN / A0A0J9XFQ4_GEOCN / A0A0J9XEC0_GEOCN / A0A0J9XK95_GEOCN	266 / 296 / 477 / 483	serine-type endopeptidase activity
Peptidase S8/S53 domain-containing protein / UniProtKB unreviewed (TrEMBL)	A0A0J9XFB1_GEOCN	915	serine-type endopeptidase activity
Phosphatidylserine decarboxylase proenzyme 2 / EC:4.1.1.65	A0A0J9XAH9_GEOCN / A0A0J9XEA1_GEOCN	950 / 1025	a 1,2-diacyl-sn-glycero-3-phospho-L-serine + H+ = a 1,2-diacyl-sn-glycero-3-phosphoethanolamine + CO2
Protease that specifically cleaves Smt3p protein conjugates / UniProtKB unreviewed (TrEMBL)	A0A0J9X786_GEOCN / A0A0J9XDV4_GEOCN	640 / 715	ubiquitin-like protein peptidase activity
ULP_PROTEASE domain-containing protein / UniProtKB unreviewed (TrEMBL)	A0A0J9X3I6_GEOCN / A0A0J9XIA4_GEOCN	2622 / 1567	ubiquitin-like protein peptidase activity
Ubiquitin-specific protease that deubiquitinates ubiquitin-protein moieties / UniProtKB unreviewed (TrEMBL)	A0A0J9X489_GEOCN / A0A0J9X8Q9_GEOCN	744 / 1341	ubiquitin-dependent protein catabolic process
Ubiquitin carboxyl-terminal hydrolase / EC:3.4.19.12	A0A0J9XB06_GEOCN / A0A0J9X414_GEOCN / A0A0J9XEK2_GEOCN / A0A0J9X501_GEOCN / A0A0J9X592_GEOCN / A0A0J9XA95_GEOCN / A0A0J9X2T2_GEOCN / A0A0J9X372_GEOCN / A0A0J9X7J1_GEOCN /A0A0J9XK21_GEOCN / A0A0J9XH58_GEOCN / A0A0J9XCS9_GEOCN	765 / 831 / 377 / 709 / 346 / 479 / 860 / 572 / 613 / 782 / 1123 / 267	Thiol-dependent hydrolysis of ester, thioester, amide, peptide and isopeptide bonds formed by the C-terminal Gly of ubiquitin
Cysteine proteinase 1 / EC:3.4.22.40	A0A0J9X6B9_GEOCN / A0A0J9XHA1_GEOCN / A0A0J9XIB3_GEOCN	445 / 455 / 515	Inactivates bleomycin B2 (a cytotoxic glycometallopeptide) by hydrolysis of a carboxyamide bond of beta-aminoalanine, but also shows general aminopeptidase activity
Possible chaperone and cysteine protease	A0A0J9XCK4_GEOCN	249	peptidase activity

/ UniProtKB unreviewed (TrEMBL)				
Calpain-like cysteine protease involved in proteolytic activation of Rim101p in response to alkaline pH / UniProtKB unreviewed (TrEMBL)		A0A0J9XGK7_GEOCN	847	calcium-dependent cysteine-type endopeptidase activity
Serine protease of SPS plasma membrane amino acid sensor system (Ssy1p-Ptr3p-Ssy5p) / UniProtKB unreviewed (TrEMBL)		A0A0J9XEA0_GEOCN / A0A0J9XD40_GEOCN	871 / 734	peptidase activity
Peptide hydrolase / EC:3.4		A0A0J9X8N9_GEOCN	396	aminopeptidase activity
Dipeptidyl peptidase 3 / EC:3.4.14.4		A0A0J9XCI8_GEOCN	688	Release of an N-terminal dipeptide from a peptide comprising four or more residues, with broad specificity. Also acts on dipeptidyl 2-naphthylamides
DUF159-domain-containing protein / UniProtKB unreviewed (TrEMBL)		A0A0J9XIK6_GEOCN	427	peptidase activity
separase / EC:3.4.22.49		A0A0J9XJN5_GEOCN	1464	All bonds known to be hydrolyzed by this endopeptidase have arginine in P1 and an acidic residue in P4. P6 is often occupied by an acidic residue or by a hydroxy-amino-acid residue, the phosphorylation of which enhances cleavage
Carboxypeptidase / EC:3.4.16		A0A0J9XAJ3_GEOCN / A0A0J9XJ85_GEOCN / A0A0J9XBC0_GEOCN / A0A0J9XKF3_GEOCN / A0A0J9X9R6_GEOCN	703 / 518 / 615 / 518 / 548	Carboxypeptidase activity
Peptide hydrolase / EC:3.4		A0A0J9XIL4_GEOCN / A0A0J9XC95_GEOCN / A0A0J9XHU1_GEOCN / A0A0J9XHY5_GEOCN / A0A0J9X468_GEOCN / A0A0J9X7M9_GEOCN	521 / 365 / 392 / 485 / 438 / 1008	aminopeptidase activity
Aminopeptidase / EC:3.4.11		A0A0J9X4K9_GEOCN / A0A0J9X3E7_GEOCN	887 / 882	aminopeptidase activity
Dipeptidyl peptidase 3 / EC:3.4.14.4		A0A0J9X8M0_GEOCN	699	aminopeptidase activity
PAN2-PAN3 deadenylation complex catalytic subunit PAN2 / EC:3.1.13.4		A0A0J9X5Y0_GEOCN	1085	Catalytic subunit of the poly(A)-nuclease (PAN) deadenylation complex, one of two cytoplasmic mRNA deadenylases involved in mRNA turnover
Metacaspase-1 / UniProtKB unreviewed (TrEMBL)		A0A0J9X509_GEOCN / A0A0J9X5P7_GEOCN	471 / 446	proteolysis
Dipeptidyl aminopeptidase / UniProtKB unreviewed (TrEMBL)		A0A0J9XA51_GEOCN	873	proteolysis

di-and tri-peptidase / UniProtKB unreviewed (TrEMBL)		A0A0J9XGA9_GEOCN / A0A0J9YHF9_GEOCN	989 / 946	di-and tri-peptidase activity
metalloprotease / UniProtKB unreviewed (TrEMBL)		A0A0J9X3T8_GEOCN / A0A0J9X7B4_GEOCN	697 /1055	metalloprotease
Vacuolar aminopeptidase yscI / UniProtKB unreviewed (TrEMBL)		A0A0J9XE48_GEOCN	526	proteolysis
Vacuolar carboxypeptidase yscS / UniProtKB unreviewed (TrEMBL)		A0A0J9XI92_GEOCN / A0A0J9XCU4_GEOCN	625 / 672	proteolysis
Cys-Gly metallo-di-peptidase / UniProtKB unreviewed (TrEMBL)		A0A0J9XFY9_GEOCN / A0A0J9XHE3_GEOCN	512 / 478	proteolysis
Glutamate carboxypeptidase II / UniProtKB unreviewed (TrEMBL)		A0A0J9X3L4_GEOCN	799	Glutamate carboxypeptidase II
Zinc metalloendopeptidase / UniProtKB unreviewed (TrEMBL)		A0A0J9XBE7_GEOCN	727	metalloprotease
Putative X-Pro aminopeptidase / UniProtKB unreviewed (TrEMBL)		A0A0J9XC47_GEOCN	465	aminopeptidase
Cytoplasmic aspartyl aminopeptidase / UniProtKB unreviewed (TrEMBL)		A0A0J9XFK2_GEOCN	489	aminopeptidase
Protein that interacts with and inhibits carboxypeptidase Y and Ira2p / UniProtKB unreviewed (TrEMBL)		A0A0J9X9L4_GEOCN	212	arboxypeptidase Y and Ira2p inhibition
Peptidase that deconjugates Smt3/SUMO-1 peptides from proteins / UniProtKB unreviewed (TrEMBL)		A0A0J9X8E3_GEOCN	794	proteolysis
Formate dehydrogenase / EC:1.17.1.9	dehydrogenases / oxidase / reductases / kinases	A0A0J9YH92_GEOCN / A0A0J9XCB4_GEOCN	362 / 362	Catalyzes the NAD+-dependent oxidation of formate to carbon dioxide
Inosine-5'-monophosphate dehydrogenase / EC:1.1.1.205		A0A0J9XK40_GEOCN / A0A0J9XCQ8_GEOCN	522 / 522	H2O + IMP + NAD+ = H+ + NADH + XMP
histidinol dehydrogenase / EC:1.1.1.23		A1X813_GEOCN	438	H2O + L-histidinol + 2 NAD+ = 3 H+ + L-histidine + 2 NADH
Glyceraldehyde-3-phosphate dehydrogenase / EC:1.2.1.12		A0A0J9XLL3_GEOCN / A0A0J9X3V9_GEOCN / A0A0J9XJM4_GEOCN	336 / 337 / 336	D-glyceraldehyde 3-phosphate + NAD+ + phosphate = (2R)-3-phospho-glyceroyl phosphate + H+ + NADH
Pentafunctional AROM polypeptide / EC:4.2.1.10		A0A0J9XAT5_GEOCN	1563	3-dehydroquinate = 3-dehydroshikimate + H2O

Succinate dehydrogenase / EC:1.3.5.1	A0A0J9XIV1_GEOCN / A0A0J9X5E8_GEOCN / A0A0J9X752_GEOCN / A0A0J9X5V6_GEOCN	272 / 270 / 641 / 176	a quinone + succinate = a quinol + fumarate
Succinate-semialdehyde dehydrogenase / EC:1.2.1.16	A0A0J9X3P0_GEOCN	496	H2O + NAD+ + succinate semialdehyde = 2 H+ + NADH + succinate
Histidine biosynthesis trifunctional protein / EC:3.5.4.19	A1BPP9_GEOCN / A0A0J9X7D2_GEOCN	844 / 866	1-(5-phospho-beta-D-ribosyl)-5'-AMP + H2O = 1-(5-phospho-beta-D-ribosyl)-5-[(5-phospho-beta-D-ribosylamino)methylideneamino]imidazole-4-carboxamide
Glutamate dehydrogenase / UniProtKB unreviewed (TrEMBL)	A0A023RBK5_GEOCN	452 / 452	Glutamate dehydrogenase
glutamate-5-semialdehyde dehydrogenase / EC:1.2.1.41	A0A0J9X8B5_GEOCN	441	L-glutamate 5-semialdehyde + NADP+ + phosphate = H+ + L-glutamyl 5-phosphate + NADPH
isocitrate dehydrogenase (NAD(+)) / EC:1.1.1.41	A0A0J9XJW9_GEOCN / A0A0J9X7C4_GEOCN	369 / 371	D-threo-isocitrate + NAD+ = 2-oxoglutarate + CO2 + NADH
Multifunctional fusion protein / EC:1.2.1.88	A0A0J9XD55_GEOCN	573	H2O + L-glutamate 5-semialdehyde + NAD+ = 2 H+ + L-glutamate + NADH
Xanthine dehydrogenase / UniProtKB unreviewed (TrEMBL)	A0A0J9XFW6_GEOCN	1365	Xanthine dehydrogenase
Aldehyde dehydrogenase / UniProtKB unreviewed (TrEMBL)	A0A0J9X635_GEOCN / A0A0J9XFR7_GEOCN / A0A0J9XBT2_GEOCN / A0A0J9X4X6_GEOCN / A0A0J9XHI0_GEOCN / A0A0J9XJ97_GEOCN / A0A0J9XE92_GEOCN A0A0J9YHL2_GEOCN	526 / 496 / 523 / 496 / 525 / 477 / 533 / 342	Aldehyde dehydrogenase
Malate dehydrogenase / EC:1.1.1.37	A0A0J9XHK6_GEOCN / A0A0J9XKL8_GEOCN / A0A0J9X9Q8_GEOCN	332 / 374 /328	(S)-malate + NAD+ = H+ + NADH + oxaloacetate
phosphoglycerate dehydrogenase / EC:1.1.1.95	A0A0J9XJ60_GEOCN	465	(2R)-3-phosphoglycerate + NAD+ = 3-phosphooxypyruvate + H+ + NADH
Proline dehydrogenase / EC:1.5.5.2	A0A0J9XDC0_GEOCN	484	a quinone + L-proline = (S)-1-pyrroline-5-carboxylate + a quinol + H+
Dihydrolipoyl dehydrogenase / EC:1.8.1.4	A0A0J9XDH1_GEOCN	502	(R)-N6-dihydrolipoyl-L-lysyl-[protein] + NAD+ = (R)-N6-lipoyl-L-lysyl-[protein] + H+ + NADH
homoserine dehydrogenase / EC:1.1.1.3	A0A0J9X4Y8_GEOCN	415	threonine biosynthetic process
Prephenate dehydrogenase [NADP(+)] / EC:1.3.1.13	A0A0J9X4C0_GEOCN	446	NADP+ + prephenate = 3-(4-hydroxyphenyl)pyruvate + CO2 + NADPH
UDP-glucose 6-dehydrogenase / EC:1.1.1.22	A0A0J9X894_GEOCN	477	H2O + 2 NAD+ + UDP-alpha-D-glucose = 3 H+ + 2 NADH + UDP-alpha-D-glucuronate

Glycerol-3-phosphate dehydrogenase / EC:1.1.5.3	A0A0J9XGN0_GEOCN / A0A0J9X5P8_GEOCN / A0A0J9X7I2_GEOCN / A0A0J9XDH2_GEOCN	619 / 617 / 369 / 370	a quinone + sn-glycerol 3-phosphate = a quinol + dihydroxyacetone phosphate
precorrin-2 dehydrogenase / EC:1.3.1.76	A0A0J9XH90_GEOCN / A0A0J9X3K1_GEOCN	276 / 522	NAD+ + precorrin-2 = 2 H+ + NADH + sirohydrochlorin
aspartate-semialdehyde dehydrogenase / EC:1.2.1.11	A0A0J9XBW1_GEOCN	361	L-aspartate 4-semialdehyde + NADP+ + phosphate = 4-phospho-L-aspartate + H+ + NADPH
3-isopropylmalate dehydrogenase / EC:1.1.1.85	A0A0J9XD73_GEOCN / A0A0J9XAW1_GEOCN	363 / 364	(2R,3S)-3-isopropylmalate + NAD+ = 4-methyl-2-oxopentanoate + CO2 + NADH
Isocitrate dehydrogenase [NADP] / EC:1.1.1.42	A0A0J9XI26_GEOCN	444	D-threo-isocitrate + NADP+ = 2-oxoglutarate + CO2 + NADPH
Homoserine dehydrogenase / EC:1.1.1.3	A0A0J9XJF3_GEOCN	362	L-homoserine + NADP+ = H+ + L-aspartate 4-semialdehyde + NADPH
Isocitrate dehydrogenase [NADP] / EC:1.1.1.42	A0A0J9X2J0_GEOCN	439	D-threo-isocitrate + NADP+ = 2-oxoglutarate + CO2 + NADPH
6-phosphogluconate dehydrogenase, decarboxylating / EC:1.1.1.44	A0A0J9XAB6_GEOCN	490	6-phospho-D-gluconate + NADP+ = CO2 + D-ribulose 5-phosphate + NADPH
S-(hydroxymethyl)glutathione dehydrogenase / EC:1.1.1.284	A0A0J9X4U2_GEOCN	378	NAD+ + S-(hydroxymethyl)glutathione = H+ + NADH + S-formylglutathione
3-hydroxybutyryl-CoA dehydrogenase / UniProtKB unreviewed (TrEMBL)	A0A0J9X3N2_GEOCN	304	oxidoreductase activity, acting on the CH-OH group of donors, NAD or NADP as acceptor
2-oxoisovalerate dehydrogenase subunit alpha / EC:1.2.4.4	A0A0J9X4Z5_GEOCN	430	(R)-N6-lipoyl-L-lysyl-[dihydrolipoyllysine-residue (2-methylpropanoyl)transferase] + 3-methyl-2-oxobutanoate + H+ = (R)-N6-(S8-2-methylpropanoyldihydrolipoyl)-L-lysyl-[dihydrolipoyllysine-residue (2-methylpropanoyl)transferase] + CO2
Glucose-6-phosphate 1-dehydrogenase / EC:1.1.1.49	A0A0J9XGH3_GEOCN / A0A0J9XHW7_GEOCN	513 / 519	D-glucose 6-phosphate + NADP+ = 6-phospho-D-glucono-1,5-lactone + H+ + NADPH
Pyruvate dehydrogenase E1 component subunit beta / EC:1.2.4.1	A0A0J9XI00_GEOCN	392	(R)-N6-lipoyl-L-lysyl-[dihydrolipoyllysine-residue acetyltransferase] + H+ + pyruvate = (R)-N6-(S8-acetyldihydrolipoyl)-L-lysyl-[dihydrolipoyllysine-residue acetyltransferase] + CO2
Acetyltransferase component of pyruvate dehydrogenase complex/ EC:2.3.1.12	A0A0J9X447_GEOCN	479	(R)-N6-dihydrolipoyl-L-lysyl-[protein] + acetyl-CoA = (R)-N6-(S8-acetyldihydrolipoyl)-L-lysyl-[protein] + CoA
Acyl-CoA dehydrogenase / UniProtKB unreviewed (TrEMBL)	A0A0J9XE58_GEOCN / A0A0J9XH16_GEOCN / A0A0J9XIU0_GEOCN / A0A0J9X374_GEOCN	442 / 613 / 442 / 417	acyl-CoA dehydrogenase activity
Acyl-CoA dehydrogenase NM	A0A0J9X7D3_GEOCN	415	Acyl-CoA dehydrogenase

domain-like protein / UniProtKB unreviewed (TrEMBL)				
Pyruvate dehydrogenase E1 component subunit alpha / EC:1.2.4.1		A0A0J9X7H5_GEOCN / A0A0J9XE67_GEOCN	389 / 412	(R)-N6-lipoyl-L-lysyl-[dihydrolipoyllysine-residue acetyltransferase] + H+ + pyruvate = (R)-N6-(S8-acetyldihydrolipoyl)-L-lysyl-[dihydrolipoyllysine-residue acetyltransferase] + CO2
Dihydroorotate dehydrogenase (quinone) / EC:1.3.5.2		M1X873_GEOCN	410	(S)-dihydroorotate + a quinone = a quinol + orotate
NADH dehydrogenase [ubiquinone] 1 alpha subcomplex subunit / UniProtKB unreviewed (TrEMBL)		A0A0J9XIW6_GEOCN / A0A0J9XBX8_GEOCN	207 / 84	mitochondrial respiratory chain complex I assembly
NAD-specific glutamate dehydrogenase / EC:1.4.1.2		A0A0J9X8G9_GEOCN / A0A0J9X9K0_GEOCN	1039 / 1046	H2O + L-glutamate + NAD+ = 2-oxoglutarate + H+ + NADH + NH4+
NADP(+)-dependent dehydrogenase / UniProtKB unreviewed (TrEMBL)		A0A0J9XFY0_GEOCN / A0A0J9XA19_GEOCN	269 / 297	NADP(+)-dependent dehydrogenase
NAD-dependent 5,10-methylenetetrahydrafolate dehydrogenase / UniProtKB unreviewed (TrEMBL)		A0A0J9X462_GEOCN	317	methylenetetrahydrofolate dehydrogenase (NADP+) activity
NAD-dependent arabinose dehydrogenase / UniProtKB unreviewed (TrEMBL)		A0A0J9XB18_GEOCN	335	D-arabinose 1-dehydrogenase [NAD(P)+] activity
NAD-dependent (R,R)-butanediol dehydrogenase / UniProtKB unreviewed (TrEMBL)		A0A0J9XAG9_GEOCN / A0A0J9XAG9_GEOCN	404 / 404	NAD-dependent (R,R)-butanediol dehydrogenase
NADPH-dependent medium chain alcohol dehydrogenase with broad substrate specificity / UniProtKB unreviewed (TrEMBL)		A0A0J9X2P3_GEOCN	349	NADPH-dependent medium chain alcohol dehydrogenase
NADPH-dependent aldehyde reductase / UniProtKB unreviewed (TrEMBL)		A0A0J9XCC0_GEOCN / A0A0J9XB22_GEOCN	350 / 350	3-beta-hydroxy-delta5-steroid dehydrogenase activity
NADH-ubiquinone oxidoreductase / UniProtKB unreviewed (TrEMBL)		A0A0J9X8Y6_GEOCN	173	NADH-ubiquinone oxidoreductase
3-methylbutanal reductase and NADPH-dependent methylglyoxal reductase (D-lactaldehyde dehydrogenase) /		A0A0J9XAL8_GEOCN/ A0A0J9X9K7_GEOCN / A0A0J9XC84_GEOCN	339 / 341 / 339	GRE2 3-methylbutanal reductase and NADPH-dependent methylglyoxal reductase

UniProtKB unreviewed (TrEMBL)				
Alpha-aminoadipate reductase / EC:1.2.1.95		A0A0J9XK20_GEOCN	1413	(S)-2-amino-6-oxohexanoate + AMP + diphosphate + NADP+ = ATP + H+ + L-2-aminoadipate + NADPH
Saccharopine dehydrogenase [NAD(+), L-lysine-forming] / EC:1.5.1.7		A0A0J9YHC2_GEOCN	368	H2O + L-saccharopine + NAD+ = 2-oxoglutarate + H+ + L-lysine + NADH
oxoglutarate dehydrogenase (succinyl-transferring) / EC:1.2.4.2		A0A0J9XJ05_GEOCN	1004	oxoglutarate dehydrogenase (succinyl-transferring)
Gluconate 5-dehydrogenase / UniProtKB unreviewed (TrEMBL)		A0A0J9XA29_GEOCN	276	oxidoreductase activity
Short-chain dehydrogenase / UniProtKB unreviewed (TrEMBL)		A0A0J9X8F9_GEOCN	277	Short-chain dehydrogenase
methylmalonate-semialdehyde dehydrogenase (CoA acylating) / EC:1.2.1.27		A0A0J9XEP4_GEOCN	542	methylmalonate-semialdehyde dehydrogenase (CoA acylating)
4-formylbenzenesulfonate dehydrogenase TsaC1/TsaC2 / UniProtKB unreviewed (TrEMBL)		A0A0J9XBY4_GEOCN	249	4-formylbenzenesulfonate dehydrogenase TsaC1/TsaC2
Dihydrolipoamide acetyltransferase component of pyruvate dehydrogenase complex / EC:2.3.1		A0A0J9X8G6_GEOCN	457	acyltransferase activity
Putative fatty aldehyde dehydrogenase / UniProtKB unreviewed (TrEMBL)		A0A0J9XJH9_GEOCN	579	oxidoreductase activity, acting on the aldehyde or oxo group of donors, NAD or NADP as acceptor
Sorbitol dehydrogenase / UniProtKB unreviewed (TrEMBL)		A0A0J9XF09_GEOCN	353	oxidoreductase activity, acting on the CH-OH group of donors, NAD or NADP as acceptor
3-ketodihydrosphingosine reductase TSC10 / EC:1.1.1.102		A0A0J9X7G8_GEOCN	311	3-dehydrosphinganine reductase
hydroxyacid-oxoacid transhydrogenase / EC:1.1.99.24		A0A0J9XDL6_GEOCN	496	(S)-3-hydroxybutanoate + 2-oxoglutarate = (R)-2-hydroxyglutarate + acetoacetate
Putative pyridoxine 4-dehydrogenase / UniProtKB unreviewed (TrEMBL)		A0A0J9XFG8_GEOCN	329	Putative pyridoxine 4-dehydrogenase
Mitochondrial alcohol dehydrogenase isozyme III /		A0A0J9XIH2_GEOCN / A0A0J9XBV1_GEOCN / A0A0J9XJK3_GEOCN	350 / 351 / 372 /	alcohol dehydrogenase isozyme III

UniProtKB unreviewed (TrEMBL)	/A0A0J9X8H3_GEOCN A0A0J9XCP6_GEOCN / A0A0J9XKV3_GEOCN	346 /350 / 350	
C-3 sterol dehydrogenase / UniProtKB unreviewed (TrEMBL)	A0A0J9X4I6_GEOCN	476	C-3 sterol dehydrogenase
Mitochondrial C1-tetrahydrofolate synthase / UniProtKB unreviewed (TrEMBL)	A0A0J9YH90_GEOCN /A0A0J9XBT0_GEOCN	977 / 939	formate-tetrahydrofolate ligase activity
Putative short-chain dehydrogenase/reductase / UniProtKB unreviewed (TrEMBL)	A0A0J9XH39_GEOCN / A0A0J9XHC9_GEOCN / A0A0J9XCW0_GEOCN	317 / 326 / 252	short-chain dehydrogenase/reductase
Protein-serine/threonine kinase / EC:2.7.11	A0A0J9XHB7_GEOCN / A0A0J9X995_GEOCN / A0A0J9X7L2_GEOCN / A0A0J9X2X3_GEOCN / A0A0J9XBU8_GEOCN	491 / 452 / 432 / 461/ 415	Protein-serine/threonine kinase
Saccharopine dehydrogenase (NADP+, L-glutamate-forming) / UniProtKB unreviewed (TrEMBL)	A0A0J9X6F8_GEOCN	450	Saccharopine dehydrogenase
dihydrolipoyllysine-residue succinyltransferase / EC:2.3.1.61	A0A0J9XGE9_GEOCN	444	dihydrolipoyllysine-residue succinyltransferase
Ketol-acid reductoisomerase, mitochondrial / EC:1.1.1.86	A0A0J9XDR2_GEOCN / A0A0J9XJN6_GEOCN	399 / 399	(2R)-2,3-dihydroxy-3-methylbutanoate + NADP+ = (2S)-2-acetolactate + H+ + NADPH
D-lactate dehydrogenase / UniProtKB unreviewed (TrEMBL)	A0A0J9X9C2_GEOCN	507	D-lactate dehydrogenase
Glycine cleavage system P protein / EC:1.4.4.2	A0A0J9X2V5_GEOCN	1017	(R)-N6-lipoyl-L-lysyl-[glycine-cleavage complex H protein] + glycine + H+ = (R)-N6-(S8-aminomethyldihydrolipoyl)-L-lysyl-[glycine-cleavage complex H protein] + CO2
Acyl-coenzyme A oxidase / EC:1.3.3.6	A0A0J9XHF1_GEOCN / A0A0J9XKH0_GEOCN	713 / 715	a 2,3-saturated acyl-CoA + O2 = a (2E)-enoyl-CoA + H2O2
2-dehydropantoate 2-reductase / EC:1.1.1.169	A0A0J9X2K9_GEOCN / A0A0J9X5C9_GEOCN / A0A0J9XDA1_GEOCN / A0A0J9XIN9_GEOCN	348 / 333 / 373 / 391	(R)-pantoate + NADP+ = 2-dehydropantoate + H+ + NADPH
Succinate semialdehyde dehydrogenase involved in the utilization of gamma-aminobutyrate (GABA) as a nitrogen source / UniProtKB unreviewed (TrEMBL)	A0A0J9XGX0_GEOCN	505	Succinate semialdehyde dehydrogenase involved in the utilization of gamma-aminobutyrate (GABA) as a nitrogen source
Fumarate reductase / EC:1.3.1.6	A0A0J9XCY8_GEOCN	471	NAD+ + succinate = fumarate + H+ + NADH
Pyrroline-5-carboxylate reductase / EC:1.5.1.2	A0A0J9X9I0_GEOCN	287	L-proline + NADP+ = 1-pyrroline-5-carboxylate + 2 H+ + NADPH

D-lactate dehydrogenase, oxidizes D-lactate to pyruvate,transcription is heme-dependent, repressed by glucose, and derepressed in ethanol or lactate / UniProtKB unreviewed (TrEMBL)		A0A0J9X9H2_GEOCN / A0A0J9XAT2_GEOCN	627 / 586	D-lactate dehydrogenase, oxidizes D-lactate to pyruvate,transcription is heme-dependent, repressed by glucose, and derepressed in ethanol or lactate
Molybdenum cofactor sulfurase / EC:2.8.1.9		A0A0J9X3K6_GEOCN	756	AH2 + L-cysteine + Mo-molybdopterin = A + H2O + L-alanine + thio-Mo-molybdopterin
non-specific serine/threonine protein kinase / EC:2.7.11.1		A0A0J9XJB2_GEOCN/ A0A0J9XES8_GEOCN	1201 / 1174	ATP + L-seryl-[protein] = ADP + H+ + O-phospho-L-seryl-[protein]
Polyprenol reductase / EC:1.3.1.94		A0A0J9X5H4_GEOCN	272	di-trans,poly-cis-dolichol + NADP+ = di-trans,cis-polyprenol + H+ + NADPH
Mitochondrial malic enzyme, catalyzes the oxidative decarboxylation of malate to pyruvate / UniProtKB unreviewed (TrEMBL)		A0A0J9XK09_GEOCN / A0A0J9XJR4_GEOCN	590 / 583	Mitochondrial malic enzyme, catalyzes the oxidative decarboxylation of malate to pyruvate
Glyoxylate reductase, acts on glyoxylate and hydroxypyruvate substrates / UniProtKB unreviewed (TrEMBL)		A0A0J9XI09_GEOCN / A0A0J9XEB9_GEOCN / A0A0J9XD49_GEOCN / A0A0J9XGB1_GEOCN	385 / 335 / 385 / 331	Glyoxylate reductase
Acetophenone reductase / UniProtKB unreviewed (TrEMBL)		M5A8V4_GEOCN	342	Acetophenone reductase
